# Supplementary material for: The Hippo pathway effector YAP inhibits NF-κB signaling and ccRCC growth by opposing ZHX2
Source: J Biol Chem. 2025 Mar 20;301(5):108430. doi: 10.1016/j.jbc.2025.108430 (PMC12018991; doi:10.1016/j.jbc.2025.108430)
Supplement: Supporting information [file mmc1.docx]

**
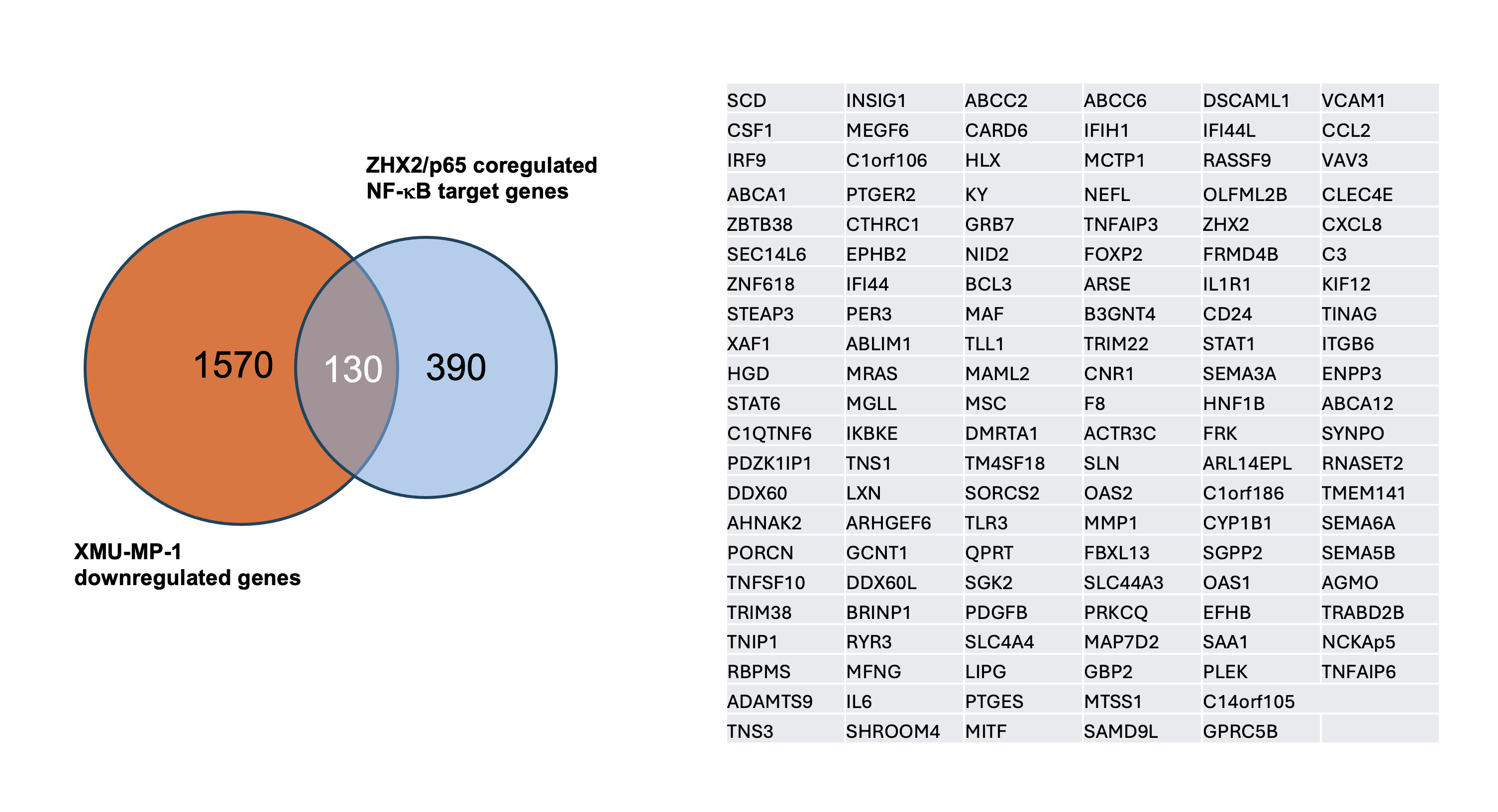
**

**Fig. S1. XMU-MP-1 inhibits NF-κB target genes co-regulated by p65 and ZHX2**

Overlap of XMU-MP1 downregulated genes and NF-kB target genes co-regulated by ZHX2 and p65 in 786-O cells (left). List of the 130 NF-kB target genes co-regulated by ZHX2 and p65 and downregulated by XMU-MP1 in 786-O cells (right).

**
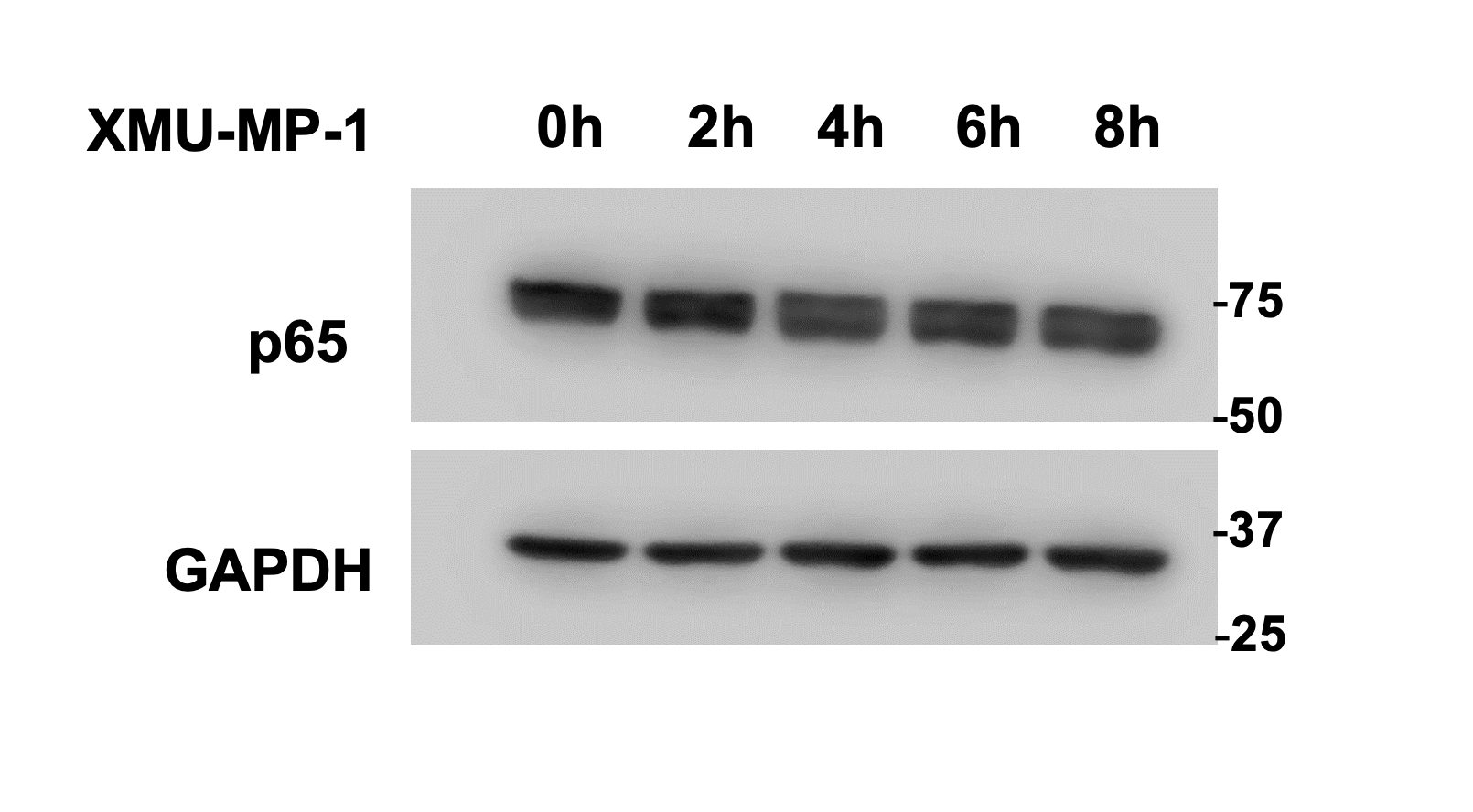
**

**Fig. S2. Hippo pathway inhibition does not affect p65 protein level**

Western blot analysis of p65 protein expression in 786-O cells treated with 2 μM XMU-MP-1 for the indicated time. GAPDH was used as a loading control.

**
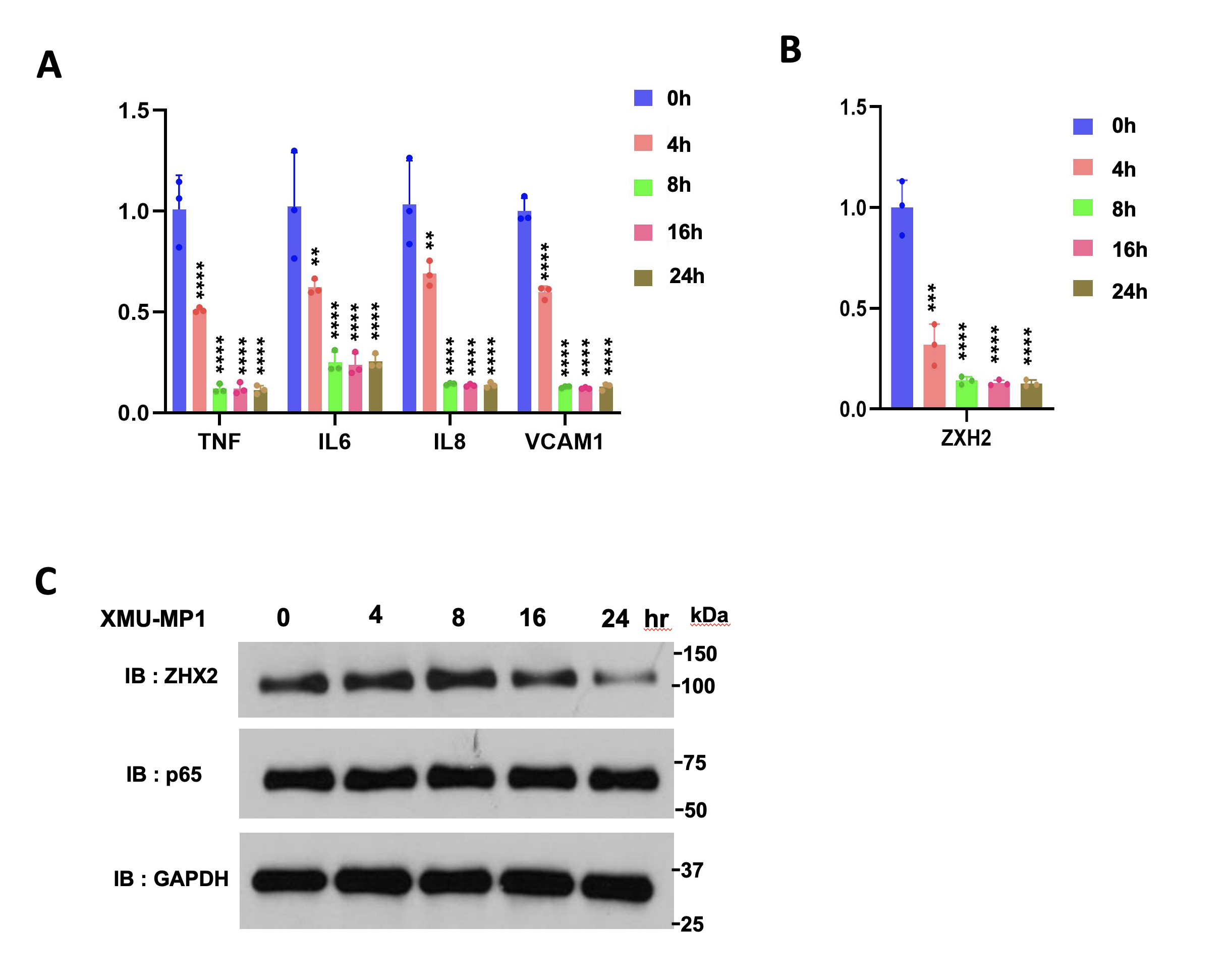
**

**Fig. S3. XMU-MP-1 inhibits NF-κB target gene expression without affecting ZHX2 protein level**

**A**-**B** Relative mRNA levels of the indicated NF-κB target genes (**A**) or *ZHX2* (**B**) in 786-O cells treated with 2 μM XMU-MP-1 for the indicated periods of time. Data in are ± SD. n=biological duplicates. ^**^P<0.01, ^***^P<0.001, ****P<0.0001 (One-way ANOVA).

**C** Western blot analysis of ZHX2 and p65 protein expression in 786-O cells treated with 2 μM XMU-MP-1 for the indicated periods of time. GAPDH was used as a loading control. ZHX2 level started to decline after XMU-MP-1 treatment for 16 hours while p65 protein level remained unchanged even after 24 hours’ treatment.
